# Addressing health challenges in rural Japan: a thematic analysis of social isolation and community solutions

**DOI:** 10.1186/s12875-024-02266-y

**Published:** 2024-01-13

**Authors:** Ryuichi Ohta, Toshihiro Yakabe, Chiaki Sano

**Affiliations:** 1 Community Care, Unnan City Hospital, 699-1221 96-1 Iida, Daito-Cho, Unnan, Shimane Prefecture Japan; 2 Department of Community Medicine Management, Faculty of Medicine, Shimane University, 89-1 Enya Cho, Izumo, Shimane Prefecture 693-8501 Japan

**Keywords:** Rural community, Sustainability, Social isolation, Physician, Help-seeking, Social cognitive theory

## Abstract

**Background:**

The establishment of sustainable connections between medical professionals and rural citizens is pivotal for effective community healthcare. Our study focuses on understanding and resolving health problems arising from social isolation, a critical barrier to achieving this goal, especially in the context of the coronavirus disease 2019(COVID-19) pandemic's impact on community dynamics respecting social cognitive theory. This study investigates the link between social isolation and rural community healthcare. We aim to develop methods that improve interaction and collaboration between healthcare providers and rural communities, ultimately enhancing the region's healthcare system.

**Methods:**

Employing thematic analysis based on social cognitive theory, we conducted semi-structured interviews with 57 community workers in rural communities. This qualitative approach enabled us to delve into the nuances of social isolation and its multifaceted impact on health and community well-being.

**Results:**

Our analysis revealed four key themes: the impact of aging on social dynamics, shifts in community relationships, unique aspects of rural community networking, and the role of these networks in driving community health. Notably, we identified specific challenges, such as the erosion of intergenerational interactions and the hesitancy to seek support, exacerbated by social isolation and negatively impacting community health.

**Conclusions:**

Our study reveals the complex factors affecting rural community sustainability, particularly social isolation influenced by privacy concerns and changing social dynamics. Emphasizing the importance of social cognitive theory, it highlights the need for adaptable healthcare systems and strong community-medical collaborations. Future research should focus on developing culturally sensitive, practical strategies for enhancing these collaborations, especially involving physicians, to address rural communities' unique challenges.

**Supplementary Information:**

The online version contains supplementary material available at 10.1186/s12875-024-02266-y.

## Background

The establishment of a trustworthy relationship between medical institutions and rural communities is crucial for effective community care. This relationship has been particularly challenged in recent times, evidenced by declining engagement and trust between rural citizens and healthcare providers [1, 2]. Studies have shown a noticeable decrease in interaction and collaboration in these areas, further compounded by the challenges of the Coronavirus Disease 2019 (COVID-19) pandemic [3, 4].

To manage people’s health effectively, it is essential to support medical institutions in providing appropriate medical care [1, 2]. The trust that people place in medical institutions is a cornerstone of their smooth functioning [3, 4]. Cooperation between community members and medical institutions is pivotal for appropriate health management, impacting the community’s overall health [5, 6]. This cooperation becomes even more vital in rural settings, where healthcare resources are often limited.

Health dialogues between medical professionals and community members can significantly improve the relationship between citizens and medical professionals. Such dialogues help clarify community needs and expectations from medical institutions, which, when addressed, increase trust and reliance on these institutions [7–9]. Moreover, making medical institutions aware of their limitations through dialogue encourages people to manage their health independently [10, 11]. Creating human connections through dialogue is essential in increasing people’s affinity for medical institutions and recognizing their necessity in community health.

The COVID-19 pandemic has significantly disrupted these essential dialogues, leading to a loss of opportunities to improve community care quality. As the world transitions towards living with the virus, it becomes imperative for community hospitals to increase opportunities for dialogue with community members to enhance medical safety and assurance [12–14]. A lack of such dialogue may contribute to social isolation in communities [15–17], which can lead to various health problems, including ineffective health-seeking behaviors and delayed use of medical resources [18, 19].

In rural communities, where healthcare resources are scarce and practical healthcare usage is crucial, social isolation can have particularly severe consequences [20, 21]. Inadequate healthcare use can significantly affect health, with the impact of social isolation varying by region [22, 23]. Post-COVID-19, an investigation into the social conditions of rural communities is necessary to address these issues [24, 25]. Furthermore, social cognitive theory can be applicable to rural health promotion based on personal and community activities [26]. Social cognitive theory can investigate personal, behavioral, and environmental factors in relation to personal and community activities. By understanding and tackling the health problems caused by social isolation through the framework of social cognitive theory, we can establish effective ways to approach and resolve community social isolation. This study investigates the link between social isolation and rural community healthcare. We aim to develop methods that improve interaction and collaboration between healthcare providers and rural communities, ultimately enhancing the region's healthcare system.

## Methods

This qualitative study used thematic analysis of participants in rural communities to investigate the health problems caused by social isolation and their solutions.

### Setting

This study was conducted in Unnan City, Shimane Prefecture, Japan. Unnan City is in the eastern part of Shimane and borders Hiroshima Prefecture to the south. Its total land area is 553.1 km^2^ and accounts for 8.3% of Shimane, most of which is covered with forests. A survey conducted in 2020 revealed that the total population of Unnan City was 36,007 (17,316 men and 18,691 women), with 40.01% of the population being older than 65 years. Unnan City has six towns (Daito, Kamo, Kisuki, Mitoya, Yoshida, and Kakeya), and each town has multifunctional autonomy and various functions in social issue management. Furthermore, 30 communities have multifunctional autonomies. In traditional protocols, multiple community groups have specific functions (e.g., community organizing, healthcare, and continual education). Thus, each autonomous community organization comprises directors, sub-directors, and clerks active in three main categories: community organizing, healthcare, and social/environmental development. Community organizing refers to citizen empowerment, effective utilization of local resources, and solving community problems through the efforts of local administrative bodies [27].

### Participants

Community workers were recruited through purposive sampling from April 2022 to March 2023. We focused on individuals actively engaged in community organizing, healthcare, and social/environmental development across Unnan City's six towns. Participants included were community workers directly involved in one of the three main categories and residing in the target towns. Those not actively engaged in community work or living outside these areas were excluded. Fifty-seven community workers participated, with 2 to 5 individuals representing each town. The participants were briefed about the study and provided informed consent to participate. The participants wore face masks, and a distance of > 2 m was maintained between them.

### Data collection

Semi-structured interviews were conducted in Japanese at each town’s community center in a private, comfortable setting. Each interview was performed face to face. The first researcher, with expertise in family medicine and public health, established rapport with participants before each interview and followed a semi-structured guide for in-depth discussions. The interview guide, developed through the discussion with the research team, included four questions: What health problems does your town face from social isolation? How does your town address community issues? How does your town manage the social isolation in the community? Do you have any ideas for improving health problems caused by social isolation? (Appendix 1) The interviewees were facilitated to discuss the interview guide as both community workers and rural citizens. Each interview lasted approximately 60 min and was recorded and transcribed verbatim.

### Reflexivity

This study was performed through interactions between researchers and participants collaboratively. The research team had diverse expertise and perspectives related to rural community care. A family physician and public health professional (RO) graduated with a master's degree in public health and family medicine and had experience in working and research in rural community health. A director of a non-profit organization (TY) worked in rural communities and has been charged with supporting isolated people in communities for 20 years. A medical educator and professor at a medical university (CS), graduated from a medical university and specialized in community health care management and education. To prevent bias, we discussed each idea about community care in analyzing the research contents of individual data analyses. We explored alternative viewpoints during the process of interpreting the data.

### Analysis

Our analysis employed inductive thematic analysis, guided by social cognitive theory, to explore health problems caused by social isolation in rural contexts and identify potential solutions. This framework, emphasizing personal, behavioral, and environmental factors related to community activities, provides a structured approach to understanding complex social phenomena, which is crucial for our study [26, 28]. Following the principles of social cognitive theory, all interviews were recorded and transcribed verbatim. This approach ensured that our data remained true to the participants' perspectives, a fundamental tenet of personal, behavioral, and environmental factors related to community activities. Initially, RO and TY carefully read the interview transcripts individually after interviewing three participants. Subsequently, the two researchers independently coded the transcripts and discussed the coding comprehensively until a mutual agreement was reached. In alignment with social cognitive theory, RO undertook a meticulous coding process, developing and revising codebooks through repeated readings of the transcripts. This iterative process is consistent with the thematic analysis framework, ensuring that our coding captures the depth and complexity of the data [26, 28]. TY independently read the content of the transcripts. After coding each participant’s data, RO and TY discussed the coding based on the codebooks until a mutual agreement was reached. The two authors introduced, merged, deleted, and refined codes, which enabled them to develop concepts and overarching themes by comparing the research materials and codes. Discussions regarding the data and coding continued until a mutual agreement was reached, and no new concepts or themes were developed. To validate our findings within the context of social cognitive theory, CS, a community care specialist, participated in discussions regarding the coding, concepts, and themes. This expert validation ensured that our interpretations were consistent with the framework and resonated with professional insights in the field. In translating the results from Japanese to English, we were careful to preserve the nuances essential to social cognitive theory, ensuring that the translation process did not compromise the integrity of our thematic analysis.

### Trustworthiness

We established credibility through prolonged engagement and iterative discussions with participants. Regular peer debriefing sessions among the research team were conducted to cross-validate findings and interpretations. Detailed descriptions of the research context, participant demographics, and the methodology are provided to allow for the assessment of transferability to other rural settings. An audit trail detailing the research process, from data collection to analysis, was maintained. This approach minimized potential researcher bias, ensuring that findings reflect the participants' experiences. The study's dependability was ensured through a consistent and transparent methodological approach. Ongoing discussions within the research team provided a reflexive critique of the process, enhancing the study's reliability.

### Ethical consideration

Participant anonymity and confidentiality were ensured throughout the study. All participants provided written informed consent before participating and answering the questionnaires. All procedures complied with the Declaration of Helsinki and its subsequent amendments. The Unnan City Hospital Clinical Ethics Committee approved the study protocol (No. 20220002).

## Results

### Results of the thematic analysis

The average age of the participants was 65.4 years (Standard deviation = 10.2), and the proportion of female participants was 49.1% (28/57). As community workers, all participants were engaged in community activities for health and the environment. Through thematic analysis, four themes were identified: social changes due to aging, relational changes in communities, community-specific networking, and connections driving community health (Table 1).

**Table 1:** Results of the thematic analysis

Theme	Concept
Social change due to aging	Dilution of human relationships
	Lack of diverse generations
	Distance to medical care facilities
Relational changes in communities	Excessive consideration of privacy
	Lack of intimacy with surroundings
	Hesitance to depend on others
Community-specific networking	Involvement of people well-known in communities
	Localization of community information
	Specific lay care in communities
Connections driving community health	Respect for community cultures
	Smooth collaboration with healthcare providers
	Active engagement of physicians

Social changes due to aging that cause social isolation include the dilution of human relationships, lack of diverse generations, and distance to medical care facilities. Relational changes in communities were explained by excessive consideration of privacy, lack of intimacy with one's surroundings, and hesitance to depend on others. Rural communities have specific networking strategies, such as the involvement of well-known people in communities, localization of community information, and specific lay care in communities. For the sustainability of rural healthcare, solving social isolation issues through connections driving community health, including respect for community culture, smooth collaboration with healthcare providers, and active engagement of physicians, were demanded.

### Social change due to aging

In rural communities, aging deterred mobility among older people. Their limited mobility hindered them from actively engaging and interacting within the communities. One participant stated, “Aging causes us to move less due to losing our driving license and few public transportation systems.” (Participant 5, 77-year-old man) Less interaction among older people was also caused by dispersed living conditions in rural areas, which diluted human relationships. One of the participants stated, “Once, I could go to other community people’s homes to talk about trivial things about our lives. Such behavior helped establish human relationships in communities. However, the lack of transportation and disparate living may inhibit us from meeting each other frequently. We may thus be reluctant to help each other." (Participant 3, 72-year-old woman) Aging and low mobility in rural areas have rendered these relationships unreliable.

The outflow of younger generations to urban areas reduced the interaction between the younger and older generations, impacting community diversity. The lack of workplaces and the need to migrate for education reduced the number of younger generations in rural communities. One participant stated, "Rural communities do not have many places to work and receive an education. Therefore, many younger generations cannot help but leave." (Participant 8, a 67-year-old man) The change in the balance between the younger and older generations has resulted in a loss of diversity in rural communities. The reality in rural contexts is the loss of mutual help between generations, which impinged older people's medical care. One of the participants stated, "Previously, younger generations supported older generations in their usual lives and visits to medical institutions. However, having fewer young people in rural areas may inhibit older people from attending medical institutions, even when they have symptoms.” (Participant 1, a 78-year-old woman) Moreover, social structural changes in rural communities have reduced the support for older people and increased the distance to medical care facilities.

### Relational changes in communities

Besides social changes associated with aging, excessive consideration of privacy has changed people's relationships in rural communities. Rural people were anxious about privacy problems; therefore, they could not communicate with people in the same community about their lives, compared with the past. One of the participants stated, "These days, dialogues about personal things might decrease. I am uncertain, but this could be affected by recent privacy issues. Many older adults may adopt excessive privacy and become reluctant to discuss personal and social issues." (Participant 11, 70-year-old woman) Rural communities have lost the opportunity to share their problems effectively.

Reduced dialogue among rural people decreased their intimacy with their surroundings. Rural residents became less aware of community issues and focused more on their issues. Eventually, they lacked interest in other community residents. One participant stated, "Recently, people in rural communities may lose interest in others because of reduced interaction. The lack of interest in others can cause various mental and health issues, especially among older people. They may lose the opportunity to receive help from others." (Participant 6, 80-year-old man) Furthermore, the reduction in mutual help in rural communities induced people's hesitance to depend on others, which is peculiar to rural communities. One participant stated, "Rural communities covered the lack of resources with mutual help. However, human relationships in communities have changed, and few people actively help each other. Therefore, they may find it difficult to obtain help." (Participant 7, 69-year-old woman).

### Community-specific networking

Rural communities have different information-sharing systems by which people share their difficulties with specific people in communities. Some people in rural communities have more information about people than others. One of the participants stated, "Rural people talk to specific people in their communities regarding their difficulties. This is because specific types of people have various social resources and connections with their stakeholders. Some of them are stakeholders themselves." (Participant 15, 81-year-old woman) People well known for their connection to social resources in communities had much information, which caused the localization of community information regarding health and social problems. One participant stated, "A few people may know most of the community issues, so they must connect various resources to social support. However, this approach may be ineffective in certain cases. This is because the number of people with social issues has increased, and these issues have varied compared with the past." (Participant 24, 71-year-old man) In rural contexts, social issues are complicated; therefore, previous methods to solve these issues have been difficult to implement.

Rural communities have mutual help established through continual community interactions. When people have health and social problems, others can help them sustain stable community conditions. One of the participants stated, "In rural communities, people have deep relationships and are dependent on others. Rural people may believe that they cannot live alone. I try to get help from other community members, and others ask me for help. We mutually help in usual cases because mutual help is vital in rural communities that lack resources." (Participant 33, 82-year-old woman) Rural people can use specific lay care services in their communities to sustain their conditions. One participant stated, "In particular, older people can get help from others to go to medical institutions when they have symptoms. They can obtain over-the-counter drugs or home remedies from community members." (Participant 18, 56-year-old woman) Establishing rural relationships among people can help sustain effective lives. However, as social conditions changed and mutual help decreased, rural residents faced more challenges. One of the participants stated, "Mutual help is vital in rural communities, but an increasing number of people here cloister themselves in their homes and confess their difficulties. These relationships need to be reconsidered in rural communities." (Participant 38, 70-year-old man) Present rural conditions are becoming unsustainable and ineffective in supporting community members.

### Connections driving community health

Respect for the present community culture has been suggested to overcome rural communities' challenges. Rural communities have undergone social changes and have faced various social problems, such as social isolation and reduced mutual help. Recognizing present rural conditions and realizing the preciousness of rural social resources was considered the standpoint for dealing with such social issues. One of the participants stated, "Social changes may not be stopped, and present trends can progress more. Present conditions should be respected for the sustainability of rural communities. Returning to the previous situation is impossible, although some may think about it. Therefore, new approaches should be applied in rural areas." (Participant 33, 68-year-old man) The exclusiveness of rural communities limits their changes. However, the participants believed that, for the sustainability of rural communities, rural citizens had to change their perceptions by collaborating with various people from outside their communities and minorities. One of the participants stated, "I think we have to change the attitudes to things outside the communities. Accepting new things can be beneficial for creating sustainable communities. An increasing number of people should be accepted into communities to activate culture and interaction." (Participant 40, 70-year-old woman) Another participant stated, “Our younger people are leaving for cities, leaving our elderly isolated and our community less vibrant.” (Participant 32, 67-year-old man) These statements highlighted the necessity of policies that address the outflow of younger individuals and consider ways to integrate diverse populations, including immigrants and minorities from different regions and countries, into the rural fabric.

Healthcare is essential for the sustainability of rural communities. Smooth collaboration with healthcare providers is critical for older rural populations. Participants hoped to connect with medical facilities more than they do now. One participant stated, "Previously, I was reluctant to communicate with other medical professionals. However, as I grew older, I needed medical care. Currently, I do not have an effective relationship with healthcare professionals. I regret my previous attitude toward healthcare." (Participant 28, a 73-year-old man) Establishing relationships with healthcare providers is vital for older adults in rural communities. However, rural communities do not collaborate with healthcare facilities, especially medical professionals. Therefore, rural stakeholders hope to collaborate with community medical professionals. One of the participants stated, "I hope to have a better relationship with medical professionals in the community. I shrink in medical institutions and cannot express my ideas." (Participant 2, a 66-year-old woman).

Active engagement of physicians in community activities is expected in rural communities. The participants expressed a sense of detachment from medical professionals, specifically from physicians. One of the participants stated, "Physicians are challenging to approach, even if I have some symptoms. I feel far from them. I go to a hospital when I have symptoms, but usually, I cannot ask them about trivial symptoms." (Participant 19, an 84-year-old woman) For effective community health, the involvement of physicians in community care and dialogue with citizens is essential. One of the participants stated, "If possible, I hope that physicians go out of hospitals and come to our communities. Initially, people in communities may be surprised, but when they can construct a relationship with physicians, they can express their anxiety and symptoms." (Participant 51, a 71-year-old man) Early detection and prevention of symptoms are essential for effective community healthcare. Establishing physician-citizen relationships and engaging physicians in community care could mitigate the limitations of citizens visiting medical care facilities for symptom prevention. One participant stated, "Older people may hesitate to visit medical care facilities despite their symptoms. When they consider physicians' friendly and open-minded engagement, they can confess their symptoms, prevent symptom progression, and continue their lives in their communities." (Participant 27, a 77-year-old woman).

## Discussion

This study, grounded in social cognitive theory, delves into the complexities of sustaining rural communities amidst social and relational changes. Social cognitive theory highlights the interplay between personal, behavioral, and environmental factors and provides a comprehensive framework for understanding these multifaceted challenges [26].

Our findings underscore that increased social isolation in rural contexts results from many social changes. These changes are supported by previous studies [29, 30] and include factors such as the migration of young people due to a lack of employment opportunities and stimulating environments [31]. This exodus alters the demographic composition and impacts the social fabric of rural communities. Such environmental shifts highlight social cognitive theory's emphasis on how external factors influence personal and collective behaviors, necessitating nuanced policy considerations [32].

Privacy issues, as revealed by our study [33, 34], pose another challenge. The need for personal privacy often conflicts with mutual support requirements in rural settings. This dichotomy adversely affects the health of rural populations [35, 36], demonstrating the theory’s assertion that personal beliefs significantly influence communal engagement and health behaviors. This suggests that interventions in rural healthcare must balance individual privacy concerns with the collective need for social support and healthcare accessibility.

Our research further identifies a significant increase in the variety and number of health issues faced by rural communities, presenting a formidable challenge to existing support systems [37]. Traditional methods, which depend heavily on specific influential community members [38, 39], may no longer suffice in the face of these evolving challenges. These observations resonate with the social cognitive framework, which posits that behavioral patterns within a community are influenced by both personal beliefs and the broader social and political context [26]. The complexity of these patterns necessitates stronger inter-community relationships and improved integration with healthcare facilities [40, 41].

The role of physicians in rural healthcare settings cannot be overstated [42, 43]. Our study highlights their critical function in enhancing health-seeking behaviors and improving health conditions in rural areas [44, 45]. This aligns with the social cognitive perspective, where physicians can serve as role models, leveraging observational learning to encourage better health practices. However, challenges remain regarding the cost-effectiveness and feasibility of increased physician involvement [46, 47]. This raises the need for innovative and economically sustainable models of healthcare delivery that can effectively leverage the influence of medical professionals in rural settings.

Moreover, the social cognitive theory underscores the importance of reciprocal determinism—the dynamic and reciprocal interaction of person, environment, and behavior [26]. In rural healthcare, this concept manifests in how individual health beliefs and practices are shaped by, and in turn, shape, the social and physical environment. For instance, the aging population in rural areas presents unique challenges [42, 43]. Aging not only impacts individual health but also influences community health needs and the demand for healthcare services [26]. Therefore, healthcare interventions in these settings must be tailored to address the specific needs of an aging population while also considering the broader social and environmental context.

Additionally, the theory’s emphasis on self-efficacy is particularly relevant. Self-efficacy, or the belief in one's ability to effect change, is a critical factor in adopting healthy behaviors. In rural communities, enhancing self-efficacy could involve community-led health initiatives and education programs that empower individuals to take an active role in their health and the health of their community [26]. Such initiatives can foster a sense of agency and collective efficacy, essential for tackling the complex health issues these communities face.

Furthermore, the theory's focus on observational learning suggests that successful health interventions in rural areas might involve leveraging local influencers and healthcare professionals as role models [26]. These individuals can demonstrate healthy behaviors and practices, thereby influencing others in the community to adopt similar behaviors [26]. This approach can be more effective, accompanied by health dialogue with family physicians in addressing the challenges posed by social isolation and the need for community solidarity in health management. The following studies should investigate the continual effects on health behaviors and quality of life of rural people [48, 49].

This study had several limitations. For credibility, triangulation was performed for thematic analysis. However, owing to the limited resources in rural contexts, only three researchers conducted this study. To improve credibility, we conducted this study over a period of 1 year. Regarding transferability, this study included various participants' descriptions but included participants from only Japanese rural communities, without maximum variation sampling. This study can be applied to rural communities with aging issues; however, further studies are required in different educational contexts. This study considered theoretical saturation, iterative data collection, and analysis to ensure dependability. However, regarding reflexivity, only three researchers with backgrounds in rural community health specialties discussed and reflected on the interview content. For better confirmation, the results of this study should be discussed with researchers in other fields.

## Conclusion

This study underscores the complex interplay of personal, behavioral, and environmental factors influencing the sustainability of rural communities. Challenges such as social isolation, heightened by privacy concerns and diminished community intimacy, highlight the need for balancing individual privacy with communal support. The evolving social and health landscape in rural areas necessitates adaptable and robust support systems capable of addressing an increasing variety of health issues. Critical to this adaptation is the establishment of collaborative relationships between rural communities and medical institutions, particularly the engagement of physicians, who play a key role in modeling healthy behaviors and influencing community health practices. Future research should focus on developing concrete, culturally sensitive methods for collaboration between rural communities and medical professionals, leveraging the strengths of social cognitive theory. Enhancing community engagement and healthcare collaboration can foster a more sustainable and healthful future for rural populations.

### Supplementary Information

**Additional file 1.**

## Data Availability

The datasets used and/or analyzed in the current study may be obtained from the corresponding author upon reasonable request.

## Abbreviations

COVID-19: Coronavirus disease 2019
HSB: Help-seeking behavior
