# Supplementary material for: Addressing health challenges in rural Japan: a thematic analysis of social isolation and community solutions
Source: BMC Prim Care. 2024 Jan 13;25:26. doi: 10.1186/s12875-024-02266-y (PMC10790262; doi:10.1186/s12875-024-02266-y)
Supplement: Supplementary file 1 — Additional file 1. [file 12875_2024_2266_MOESM1_ESM.docx]

**Interview guide**

**Inquiring about health problems caused by social isolation and their solutions in rural communities.**

We would like to interview you to investigate rural health problems by social isolation and their solutions.

**The explanation**

-The interview’s duration is about 60 minutes.

-The contents of the interview were used in this research.

-The interview contents are used for research only.

-The content of the interview will be recorded and transcribed verbatim. The recorded data will be discarded after use to protect personal information.

-If you are inconvenienced, you can withdraw at any time and will not suffer any medical disadvantage.

-In the unlikely event that you suffer a disadvantage, you will immediately stop participating in the research and not use the data.

-Participants' information is based on national guidelines, including that privacy and human rights are adequately protected. If you have any questions or concerns regarding this request, please contact the following: Unnan City Hospital Community Care Department: +81854-47-7500

**Interview guide**

I will ask the following four questions:

1. What health problems does your town face owing to social isolation?
2. How does your town address community issues?
3. How does your town manage the social isolation in the community?
4. Do you have any ideas for improving health problems caused by social isolation?

Ryuichi Ohta

Unnan city hospital
